# Supplementary material for: In vitro pharmacokinetics and pharmacodynamics of the diarylquinoline TBAJ-587 and its metabolites against Mycobacterium tuberculosis
Source: Front Microbiol. 2026 Jun 29;17:1828998. doi: 10.3389/fmicb.2026.1828998 (PMC13357423; doi:10.3389/fmicb.2026.1828998)
Supplement: Supplementary file 1 [file Table_1.DOCX]

**SUPPLEMENTARY MATERIALS**

***In vitro* pharmacokinetics and pharmacodynamics of the diarylquinoline TBAJ-587 and its metabolites against *Mycobacterium tuberculosis***

Diana Angelica Aguilar-Ayala^1^, Marie Sylvianne Rabodoarivelo^1^, Maxime R. Eveque-Mourroux^2, #^, Albin AM Leding^3^, Lindsay Sonnenkalb^4^, Ana Picó Marco^1^, Nicolas Willand^2^, Natalya Serbina^5^, Ulrika SH Simonsson^3^, Ainhoa Lucía^1, 6^ and Santiago Ramón-García^1, 6, 7^, on behalf of the ERA4TB consortium.

^1^Department of Microbiology, Pediatrics, Radiology and Public Health, Faculty of Medicine, University of Zaragoza, Spain.

^2^Univ. Lille, Inserm, Institut Pasteur Lille, U1177 - Drugs and Molecules for living

Systems, F-59000 Lille, France.

^3^Dept. of Pharmaceutical Biosciences, Uppsala University, Uppsala, Sweden.

^4^Molecular and Experimental Mycobacteriology, Research Center Borstel Leibniz Lung Center, Parkallee 1-40, 23845, Borstel, Germany.

^5^The Global Alliance for TB Drug Development, New York, NY, United States.

^6^Spanish Network for Research on Respiratory Diseases (CIBERES), Carlos III Health Institute, Madrid, Spain.

^7^Research & Development Agency of Aragón Foundation (Fundación ARAID), Zaragoza, Spain.

^#^Current address: Metabolomics & Analytics Centre. Leiden Academic Centre for Drug Research. Leiden University, 2300RA Leiden, The Netherlands.

**Corresponding author:** Santiago Ramón-García. Email: santiramon@unizar.es

**Running title (52/54 characters):** *In vitro* PKPD of TBAJ-587 for Tuberculosis treatment

**Keywords:** tuberculosis, TBAJ-587, pharmacokinetics/pharmacodynamics, time-kill assay.

**SUPPLEMENTARY TABLES**

**Table S1. Time-kill assays conditions for TBAJ-587 and its main metabolites against *M. tuberculosis* H37Rv.**

| **Condition Num.** | **Drug** | **Media** | **Concentration (mg/L)** | **Specifications** |
| --- | --- | --- | --- | --- |
| 1/2/3 | TBAJ587 | ST/CHO/FA | 0,0016 | Ca. 1/20x MIC |
| 4/5/6 | TBAJ587 | ST/CHO/FA | 0,006 | Ca. 1/5x MIC |
| 7/8/9 | TBAJ587 | ST/CHO/FA | 0,016 | Ca. 1/2x MIC |
| 10/11/12 | TBAJ587 | ST/CHO/FA | 0,031 | Ca. 1x MIC |
| 13/14/15 | TBAJ587 | ST/CHO/FA | 0,063 | Ca. 2x MIC |
| 16/17/18 | TBAJ587 | ST/CHO/FA | 0,156 | Ca. 5x MIC |
| 19/20/21 | TBAJ587 | ST/CHO/FA | 0,625 | Ca. 20x MIC |
| 22/23/24 | TBAJ587 | ST/CHO/FA | 3,13 | Ca. 100x MIC |
| 25/26/27 | TBAJ587 | ST/CHO/FA | 9,38 | Ca. 300x MIC |
| 28/29/30 | M2 | ST/CHO/FA | 0,02 | Ca. 1/20x MIC |
| 31/32/33 | M2 | ST/CHO/FA | 0,08 | Ca. 1/5x MIC |
| 34/35/36 | M2 | ST/CHO/FA | 0,2 | Ca. 1/2x MIC |
| 37/38/39 | M2 | ST/CHO/FA | 0,4 | Ca. 1x MIC |
| 40/41/42 | M2 | ST/CHO/FA | 0,8 | Ca. 2x MIC |
| 43/44/45 | M2 | ST/CHO/FA | 2 | Ca. 5x MIC |
| 46/47/48 | M2 | ST/CHO/FA | 8 | Ca. 20x MIC |
| 49/50/51 | M2 | ST/CHO/FA | 40 | Ca. 100x MIC |
| 52/53/54 | M2 | ST/CHO/FA | 120 | Ca. 300x MIC |
| 55/56/57 | M3 | ST/CHO/FA | 0,006 | Ca. 1/20x MIC |
| 58/59/60 | M3 | ST/CHO/FA | 0,025 | Ca. 1/5x MIC |
| 61/62/63 | M3 | ST/CHO/FA | 0,063 | Ca. 1/2x MIC |
| 64/65/66 | M3 | ST/CHO/FA | 0,13 | Ca. 1x MIC |
| 67/68/69 | M3 | ST/CHO/FA | 0,25 | Ca. 2x MIC |
| 70/71/72 | M3 | ST/CHO/FA | 0,63 | Ca. 5x MIC |
| 73/74/75 | M3 | ST/CHO/FA | 2,5 | Ca. 20x MIC |
| 76/77/78 | M3 | ST/CHO/FA | 12,5 | Ca. 100x MIC |
| 79/80/81 | M3 | ST/CHO/FA | 37,5 | Ca. 300x MIC |
| 82/83/84 | M12 | ST/CHO/FA | 0,23 | Ca. 1/20x MIC |
| 85/86/87 | M12 | ST/CHO/FA | 0,9 | Ca. 1/5x MIC |
| 88/89/90 | M12 | ST/CHO/FA | 2,25 | Ca. 1/2x MIC |
| 91/92/93 | M12 | ST/CHO/FA | 4,5 | Ca. 1x MIC |
| 94/95/96 | M12 | ST/CHO/FA | 9 | Ca. 2x MIC |
| 97/98/99 | M12 | ST/CHO/FA | 22,5 | Ca. 5x MIC |
| 100/101/102 | M12 | ST/CHO/FA | 90 | Ca. 20x MIC |
| 103/104/105 | M12 | ST/CHO/FA | 450 | Ca. 100x MIC |
| 106/107/108 | M12 | ST/CHO/FA | 1350 | Ca. 300x MIC |
| 109/110/111 | None | ST/CHO/FA | No drug | Growth controls |

**Table S2. Minimum inhibitory concentrations of TBAJ-587 and its main metabolites against *M. tuberculosis* H37Rv Pasteur under eight culture conditions.** See table note below for MIC definition and replicate details.

| **Compound/ Medium** | **Biological replicate** | **MIC (mg/L)** | | | | | | | |
| --- | --- | --- | --- | --- | --- | --- | --- | --- | --- |
|  |  | **Standard** | **Cholesterol + tyloxapol** | **Standard + tyloxapol** | **Fatty acids** | **Glucose** | **Pyruvate** | **Acetate** | **Butyrate** |
| TBAJ587 | 1 | 0.062  0.062  0.031 | 0.031  0.031  0.031 | 0.062  0.062  0.062 | 0.062  0.062  0.031 | 0.031  0.031  0.062 | 0.031  0.062  0.062 | 0.031  0.031  0.031 | 0.062  0.062  0.031 |
|  | 2 | 0.062  0.062  0.031 | 0.031  0.031  0.031 |  | 0.031  0.031  0.031 |  |  |  |  |
|  | 3 | 0.062  0.031  0.031 |  |  |  |  |  |  |  |
| M2 | 1 | 0.4  0.4  0.2 | 0.8  0.8  0.4 | 0.8  0.8  1.6 | 0.8  0.4  0.4 | 0.4  0.4  0.4 | 0.4  0.8  0.8 | 0.8  0.8  0.8 | 0.2  0.2  3.2 |
|  | 2 | 0.4  0.4  3.2 | 0.8  1.6  1.6 |  | 0.4  0.4  0.4 |  |  |  |  |
|  | 3 | 0.4  0.4  0.4 |  |  |  |  |  |  |  |
| M3 | 1 | 0.125  0.062  0.062 | 0.125  0.062  0.062 | 0.125  0.125  0.125 | 0.062  0.125  0.125 | 0.062  0.062  0.125 | 0.062  0.062  0.125 | 0.031  0.031  0.031 | 0.062  0.062  0.062 |
|  | 2 | 0.125  0.125  0.125 | 0.125  0.062  0.062 |  | 0.062  0.062  0.125 |  |  |  |  |
|  | 3 | 0.125  0.125  0.062 |  |  |  |  |  |  |  |
| M12 | 1 | >2.25  >2.25  >2.25 | >2.25  >2.25  >2.25 | >2.25  >2.25  >2.25 | >2.25  >2.25  2.25 | 2.25  2.25  >2,25 | 2.25  2.25  2.25 | >2.25  >2.25  >2.25 | 1.125  1.125  2.25 |
|  | 2 | 4.5  2.25  2.25 | 9  9  9 |  | 9  2.2  2.25 |  |  |  |  |
|  | 3 | 4.5  4.5  4.5 |  |  |  |  |  |  |  |
| BDQ | 1 | 0.125  0.25  0.25 | 0.062  0.062  0,125 | 0.125  0.125  0.125 | 0.062  0.125  0.125 | 0.125  0.25  0.25 | 0.031  0.062  0.062 | 0.25  0.25  0.25 |  |
| MXF | 1 | 0.125  0.125  0.25 | 0.062  0.062  0.062 | 0.062  0.062  0.125 | 0.062  0.062  0.125 | 0.062  0.125  0.25 | 0.125  0.125  0.125 | 0.125  0.125  0.125 |  |
| LZD | 1 | 0.5  1  2 | 2  2  2 | 2.0  2.0  2.0 | 1  1  2 | 1  1  2 | 1  2  2 | 2  2  2 |  |

MIC values are expressed in mg/L and correspond to the lowest concentration meeting an ≥80% inhibition threshold, based on resazurin fluorescence normalized to the untreated growth control. TBAJ-587 and its metabolites were tested in at least two biological replicates in standard, cholesterol + tyloxapol, and fatty acids media; all other determinations, including comparator drugs used as internal assay controls (BDQ, MXF, LZD), were performed in one biological replicate. Each biological replicate included three technical replicates. When MIC values varied across replicates, the most frequent observed MIC value was used to select concentrations for subsequent time kill assays. The concentration range tested for TBAJ-587, M3 and BDQ was 0.002 - 0.25 mg/L. The range for M2 metabolite was 0.012 – 1.6 mg/L. Two ranges were tested for M12: 0.017 – 2.25 mg/L and 0.017 - 18 mg/L.

**Table S3. Whole Genome Sequencing data of the TKA regrowth isolates.** See table footnote for variant selection criteria and column definition.

| **Strain ID** | **Org ID** | **Library name** | **#Pos.** | **Ref** | **Alternative allele** | **Relevance** | **Category** | **Gene** | **Gene Name** | **Type** | **CovFor** | **CovRev** | **Qual 20** | **Freq** | **Cov** | **Subst.** |
| --- | --- | --- | --- | --- | --- | --- | --- | --- | --- | --- | --- | --- | --- | --- | --- | --- |
| 1 | **10 ST** | ERC-216 | 779131 | C | GAP | resistant | nonessential | Rv0678 | - | Del | 23 | 20 | 43 | 43,9 | 98 | 142 del c |
| 1 | **10 ST** | ERC-216 | 779165 | C | GAP | resistant | nonessential | Rv0678 | - | Del | 9 | 5 | 14 | 14,9 | 94 | 176 del c |
| 1 | **10 ST** | ERC-216 | 779450 | T | C | resistant | nonessential | Rv0678 | - | SNP | 15 | 8 | 23 | 24,2 | 95 | L154P (ctg/cCg) |
| 1 | **10 ST** | ERC-216 | 1474955 | T | C | unknown | - | Rvnr02 | rrl | SNP | 3 | 9 | 12 | 27,3 | 44 | - |
| 1 | **10 ST** | ERC-216 | 2210336 | T | G | unknown | nonessential | Rv1966 | mce3A | SNP | 2 | 7 | 9 | 11,8 | 76 | L337R (ctg/cGg) |
| 1 | **10 ST** | ERC-216 | 2210339 | C | A | unknown | nonessential | Rv1966 | mce3A | SNP | 2 | 7 | 9 | 12,2 | 74 | P338Q (ccg/cAg) |
| 1 | **10 ST** | ERC-216 | 4229807 | A | G | unknown | nonessential | Rv3783 | rfbD | SNP | 6 | 6 | 12 | 12,0 | 100 | T184A (acc/Gcc) |
| 1 | **10 ST** | ERC-216 | 4229813 | T | C | unknown | nonessential | Rv3783 | rfbD | SNP | 6 | 6 | 12 | 11,5 | 104 | Y186H (tac/Cac) |
| 2 | **11 CHO** | ERC-217 | 779041 | G | A | *low-freq. resistance associated | nonessential | Rv0678 | - | Ins | 3 | 3 | 6 | 6,7 | 6 | 52 ins a |
| 2 | **11 CHO** | ERC-217 | 779168 | T | C | *low-freq. resistance associated | nonessential | Rv0678 | - | SNP | 5 | 2 | 7 | 7,3 | 96 | L60P (ctg/cCg) |
| 2 | **11 CHO** | ERC-217 | 779191 | A | G | resistant | nonessential | Rv0678 | - | SNP | 10 | 10 | 20 | 21,3 | 94 | S68G (agc/Ggc) |
| 2 | **11 CHO** | ERC-217 | 779292 | G | GAP | *low-freq. resistance associated | nonessential | Rv0678 | - | Del | 3 | 3 | 6 | 7,0 | 86 | 303 del g |
| 2 | **11 CHO** | ERC-217 | 1460745 | C | G | resistant | essential | Rv1304 | atpB | SNP | 10 | 8 | 18 | 23,1 | 78 | L168V (ctc/Gtc) |
| 2 | **11 CHO** | ERC-217 | 2210336 | T | G | unknown | nonessential | Rv1966 | mce3A | SNP | 3 | 11 | 14 | 17,3 | 81 | L337R (ctg/cGg) |
| 2 | **11 CHO** | ERC-217 | 2210339 | C | A | unknown | nonessential | Rv1966 | mce3A | SNP | 3 | 11 | 14 | 17,9 | 78 | P338Q (ccg/cAg) |
| 2 | **11 CHO** | ERC-217 | 779035-6 | AT | GAP | resistant | nonessential | Rv0678 | - | Del | 7 | 5 | 16 | 14,8 | 81 | 46 del at |
| 3 | **12 FA** | ERC-218 | 234475 | T | G | unknown | nonessential | Rv0197 | - | SNP | 4 | 2 | 6 | 20,0 | 30 | Y749D (tat/Gat) |
| 3 | **12 FA** | ERC-218 | 234476 | A | C | unknown | nonessential | Rv0197 | - | SNP | 4 | 2 | 6 | 15,8 | 38 | Y749S (tat/tCt) |
| 3 | **12 FA** | ERC-218 | 234478 | C | A | unknown | nonessential | Rv0197 | - | SNP | 4 | 2 | 6 | 15,0 | 40 | P750T (ccc/Acc) |
| 3 | **12 FA** | ERC-218 | 779191 | A | g | *low-freq. resistance associated | nonessential | Rv0678 | - | SNP | 1 | 1 | 2 | 2,5 | 80 | S68G (agc/Ggc) |
| 3 | **12 FA** | ERC-218 | 779363 | T | C | *low-freq. resistance associated | nonessential | Rv0678 | - | SNP | 1 | 2 | 3 | 5,5 | 55 | L125P (ctg/cCg) |
| 3 | **12 FA** | ERC-218 | 779447 | C | A | *low-freq. resistance associated | nonessential | Rv0678 | - | SNP | 1 | 4 | 5 | 7,9 | 63 | A153D (gcc/gAc) |
| 3 | **12 FA** | ERC-218 | 3068836 | C | T | unknown | nonessential | Rv2756c | hsdM | SNP | 4 | 4 | 8 | 12,9 | 62 | R416R (cgg/cgA) |
| 4 | **13 ST** | ERC-219 | 234475 | T | G | unknown | nonessential | Rv0197 | - | SNP | 3 | 4 | 7 | 19,4 | 36 | Y749D (tat/Gat) |
| 4 | **13 ST** | ERC-219 | 234476 | A | C | unknown | nonessential | Rv0197 | - | SNP | 3 | 4 | 7 | 16,7 | 42 | Y749S (tat/tCt) |
| 4 | **13 ST** | ERC-219 | 234478 | C | A | unknown | nonessential | Rv0197 | - | SNP | 4 | 4 | 8 | 22,2 | 36 | P750T (ccc/Acc) |
| 4 | **13 ST** | ERC-219 | 649106 | T | A | unknown | essential | Rv0557 | mgtA | SNP | 2 | 4 | 6 | 10,0 | 60 | S191T (tcg/Acg) |
| 4 | **13 ST** | ERC-219 | 779053 | C | T | *low-freq. resistance associated | nonessential | Rv0678 | - | SNP | 5 | 2 | 7 | 8,0 | 87 | Q22_ (cag/Tag) |
| 4 | **13 ST** | ERC-219 | 906993 | G | A | unknown | nonessential | Rv0812 | - | SNP | 4 | 4 | 8 | 10,1 | 79 | A191T (gcc/Acc) |
| 4 | **13 ST** | ERC-219 | 958563 | A | G | unknown | nonessential | Rv0861c | ercc3 | SNP | 3 | 3 | 6 | 13,3 | 45 | I530T (atc/aCc) |
| 4 | **13 ST** | ERC-219 | 990399 | A | T | unknown | nonessential | Rv0890c | - | SNP | 3 | 4 | 7 | 14,0 | 50 | L733Q (ctg/cAg) |
| 4 | **13 ST** | ERC-219 | 1461240 | A | C | resistant | essential | Rv1305 | atpE | SNP | 10 | 6 | 16 | 25,4 | 63 | I66L (atc/Ctc) |
| 4 | **13 ST** | ERC-219 | 1837303 | A | C | unknown | nonessential | Rv1633 | uvrB | SNP | 3 | 3 | 6 | 11,5 | 52 | N77H (aac/Cac) |
| 4 | **13 ST** | ERC-219 | 4400580 | A | G | unknown | nonessential | Rv3911 | sigM | SNP | 2 | 2 | 4 | 10,3 | 39 | Q132R (cag/cGg) |
| 4 | **13 ST** | ERC-219 | 779411-2 | AT | GAP | resistant | nonessential | Rv0678 | - | Del | 24 | 26 | 50 | 62,5 | 80 | 422 del at |
| 5 | **14 CHO** | ERC-220 | 779047 | G | T | *low-freq. resistance associated | nonessential | Rv0678 | - | SNP | 3 | 4 | 7 | 9,2 | 76 | V20F (gtc/Ttc) |
| 5 | **14 CHO** | ERC-220 | 779192 | G | C | resistant | nonessential | Rv0678 | - | Ins | 9 | 7 | 16 | 17,8 | 16 | 203 ins gg |
| 5 | **14 CHO** | ERC-220 | 779216 | A | GAP | resistant | nonessential | Rv0678 | - | Del | 9 | 5 | 15 | 14,7 | 95 | 227 del a |
| 5 | **14 CHO** | ERC-220 | 779414 | T | G | *low-freq. resistance associated | nonessential | Rv0678 | - | SNP | 2 | 3 | 5 | 5,1 | 98 | L142R (ctg/cGg) |
| 5 | **14 CHO** | ERC-220 | 1870075 | C | A | unknown | essential | Rv1656 | argF | SNP | 5 | 5 | 10 | 11,5 | 87 | R52S (cgc/Agc) |
| 5 | **14 CHO** | ERC-220 | 2181393 | T | A | unknown | nonessential | Rv1929c | - | SNP | 2 | 2 | 4 | 16,7 | 24 | T172S (acg/Tcg) |
| 5 | **14 CHO** | ERC-220 | 3391069 | G | A | unknown | essential | Rv3031 | - | SNP | 6 | 2 | 8 | 12,1 | 66 | G383E (gga/gAa) |
| 5 | **14 CHO** | ERC-220 | 3391072 | T | C | unknown | essential | Rv3031 | - | SNP | 6 | 2 | 8 | 11,6 | 69 | F384S (ttc/tCc) |
| 5 | **14CHO** | ERC-220 | 4128130 | T | A | unknown | nonessential | Rv3685c | cyp137 | SNP | 7 | 2 | 9 | 10,8 | 83 | D199V (gac/gTc) |
| 6 | **15FA** | ERC-221 | 779234 | G | T | resistant | nonessential | Rv0678 | - | SNP | 58 | 44 | 102 | 88,7 | 115 | R82L (cgg/cTg) |

Variants were selected according to the following criteria: location in non-repetitive coding genes, support by reads in both forward and reverse orientations, and non-synonymous SNPs resulting in missense mutations. Intergenic variants and synonymous SNPs were excluded. Column content: **Strain ID** and **Org ID** stand for the isolate identifier as in Table S3; **Library name**, sequencing library name as deposited at NCBI under the BioProject accession number PRJNA1435615**; #Pos.,** nucleotide position in the reference genome; **Ref**, reference allele; **Alternative allele** refers to the mutated allele; **Relevance**, relevance to bedaquiline resistance; **Category,** functional category of the affected gene; **Gene/Gene** **name,** gene identifier and name; **Type,** variant type; **Cov-For/CovRev,** forward and reverse coverage; **Qual 20,** quality score; **Freq,** variant frequency; **Cov,** total coverage; and **Subst.,** nucleotide or amino acid substitution.

**Table S4. Ratios of the actual exposure relative to expected exposure of TBAJ-587 and its main metabolites.** Exposure based on the area under the concentration-time (AUC/T) curve from either measured concentration over time (actual exposure) or the expected concentrations over time (added concentration in culture flasks, expected exposure). AUC ratios with values close to 1 indicate no meaningful differences in PKPD performance between the actual and the expected drug exposures. AUC rations below 1 indicate that actual drug exposures were much lower than expected.

$Relative exposure = \frac{AUC\_Actual\_exposure}{AUC\_Expected\_exposure}$

ST: standard broth culture, CHO: cholesterol broth culture, FA: oleic, palmitic, and stearic fatty acids broth culture.

| **Compound** | **ST** | **CHO** | **FA** |
| --- | --- | --- | --- |
| **TBAJ-587** | 0.05 | 0.71 | 0.06 |
| **M2** | 0.80 | 1.59 | 0.65 |
| **M3** | 0.15 | 0.56 | 0.07 |
| **M12** | 0.69 | 1.58 | 1.05 |

**SUPPLEMENTARY FIGURES**

**Figure S1. Drug concentration of TBAJ-587 in standard broth.** Relative concentration to time zero of TBAJ-587 in standard medium for the time kill assay, dotted line marks the baseline concentration. Below quantification limit marked on x-axis.


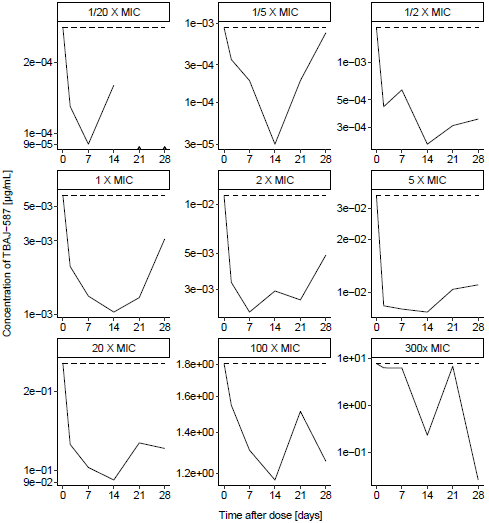


**Figure S2. Drug concentration of TBAJ-587 in cholesterol broth.** Relative concentration to time zero of TBAJ-587 in cholesterol medium for the time kill assay, dotted line marks the baseline concentration. Below quantification limit marked on x-axis.


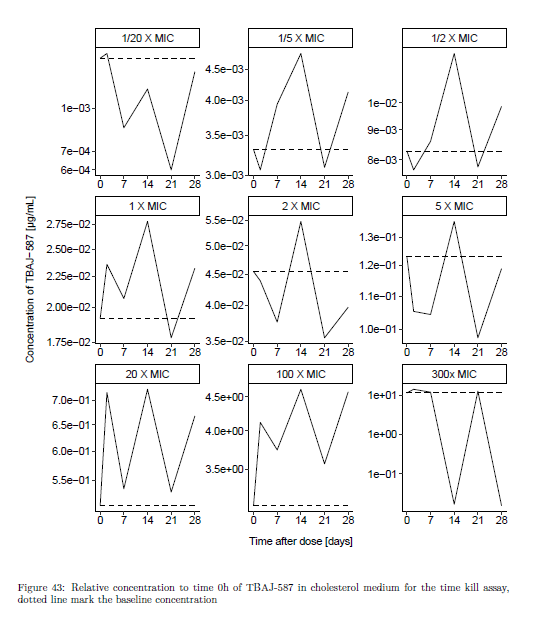


**Figure S3. Drug concentration of TBAJ-587 in fatty acids broth.** Relative concentration to time zero of TBAJ-587 in fatty acids medium for the time kill assay, dotted line marks the baseline concentration. Below quantification limit marked on x-axis.


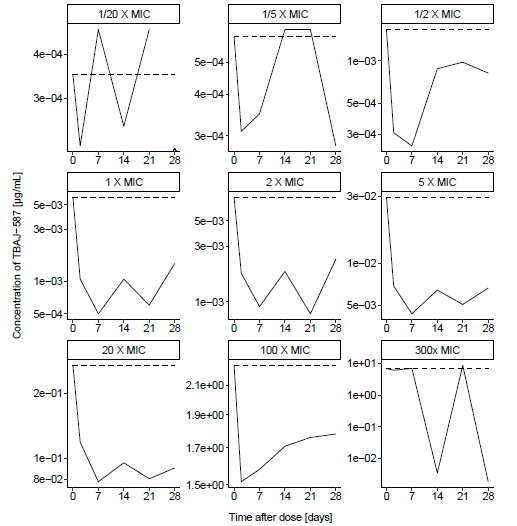


**Figure S4. Drug concentration of M2 in standard broth.** Relative concentration to time zero of M2 in standard medium for the time kill assay, dotted line marks the baseline concentration. Below quantification limit marked on x-axis.


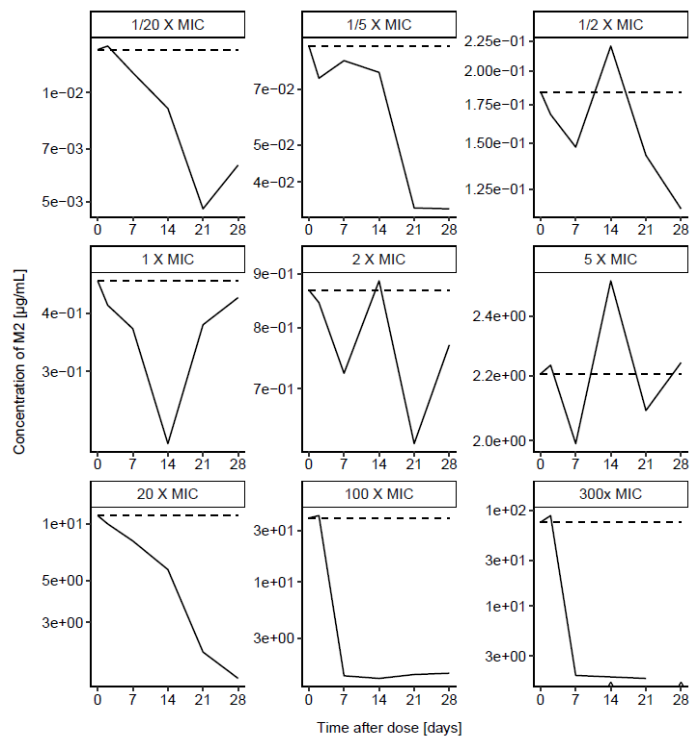


**Figure S5. Drug concentration of M2 in cholesterol broth.** Relative concentration to time zero of M2 in cholesterol medium for the time kill assay, dotted line marks the baseline concentration. Below quantification limit marked on x-axis.


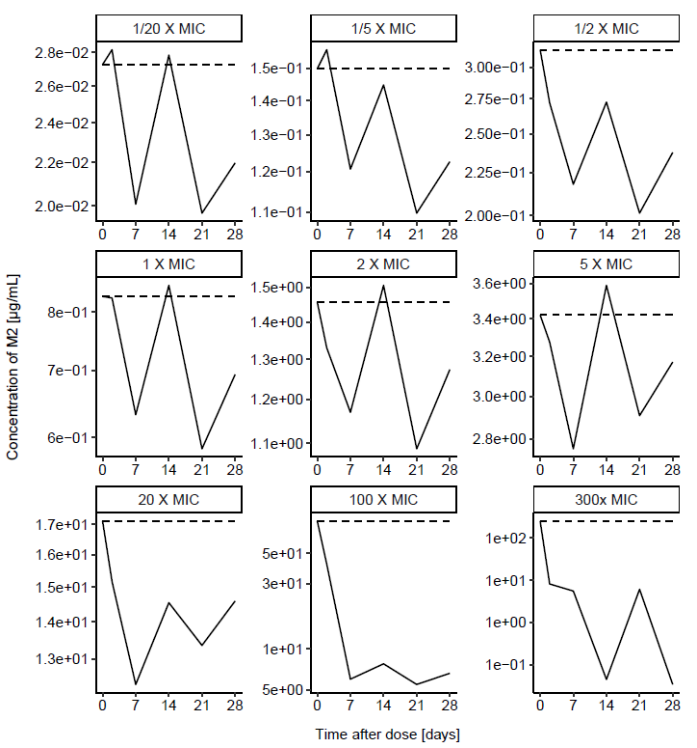


**Figure S6. Drug concentration of M2 in fatty acids broth.** Relative concentration to time zero of M2 in fatty acids medium for the time kill assay, dotted line marks the baseline concentration. Below quantification limit marked on x-axis.


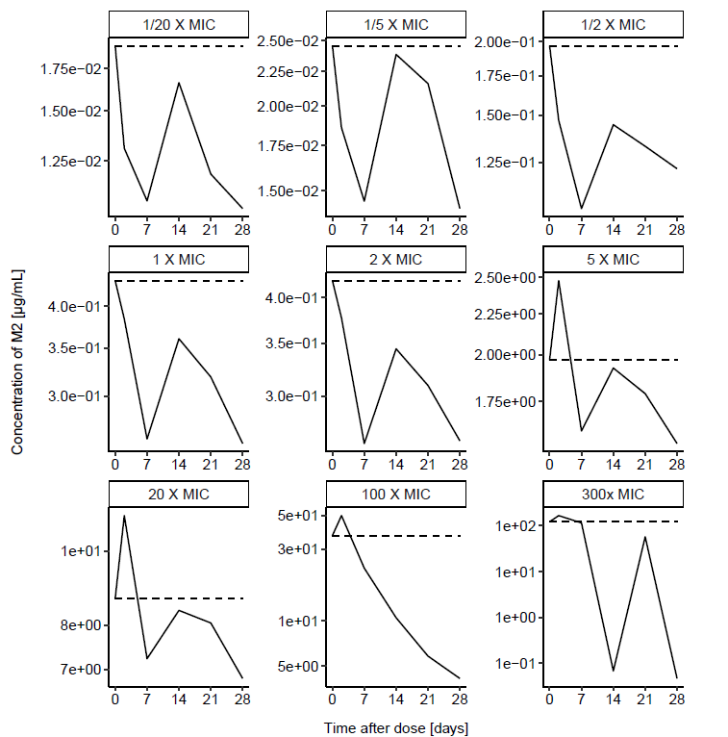


**Figure S7. Drug concentration of M3 in standard broth.** Relative concentration to time zero of M3 in standard medium for the time kill assay, dotted line marks the baseline concentration. Below quantification limit marked on x-axis.


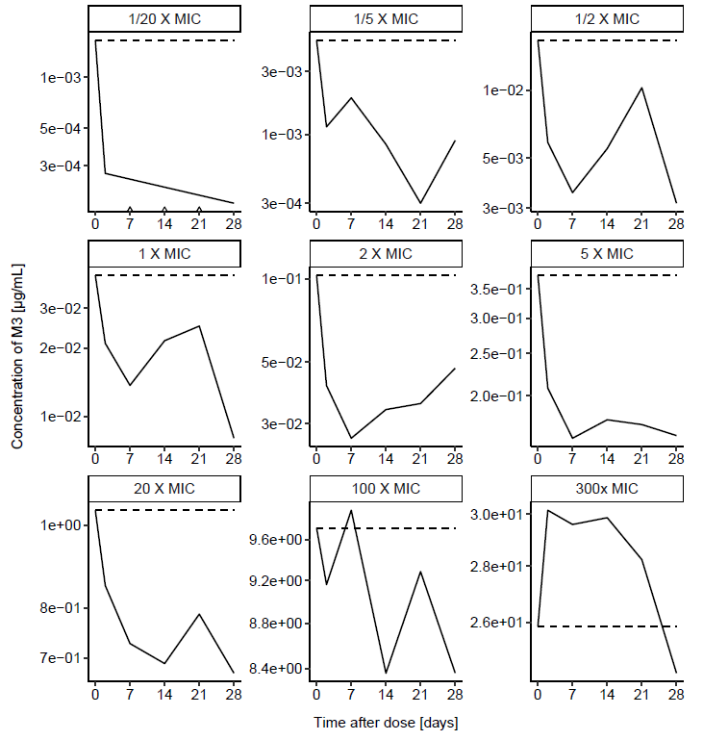


**Figure S8. Drug concentration of M3 in cholesterol broth.** Relative concentration to time zero of M3 in cholesterol medium for the time kill assay, dotted line marks the baseline concentration. Below quantification limit marked on x-axis.


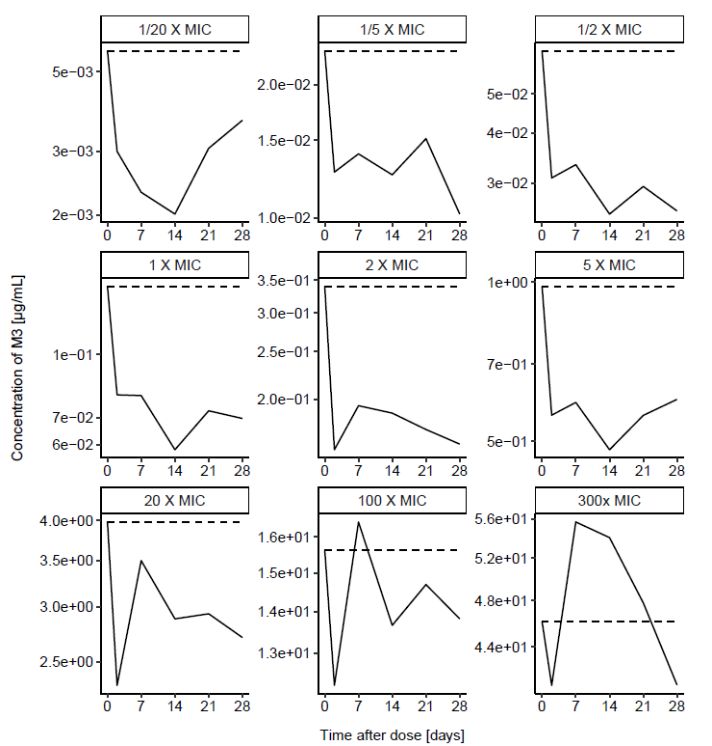


**Figure S9. Drug concentration of M3 in fatty acids broth.** Relative concentration to time zero of M3 in fatty acids medium for the time kill assay, dotted line marks the baseline concentration. Below quantification limit marked on x-axis.


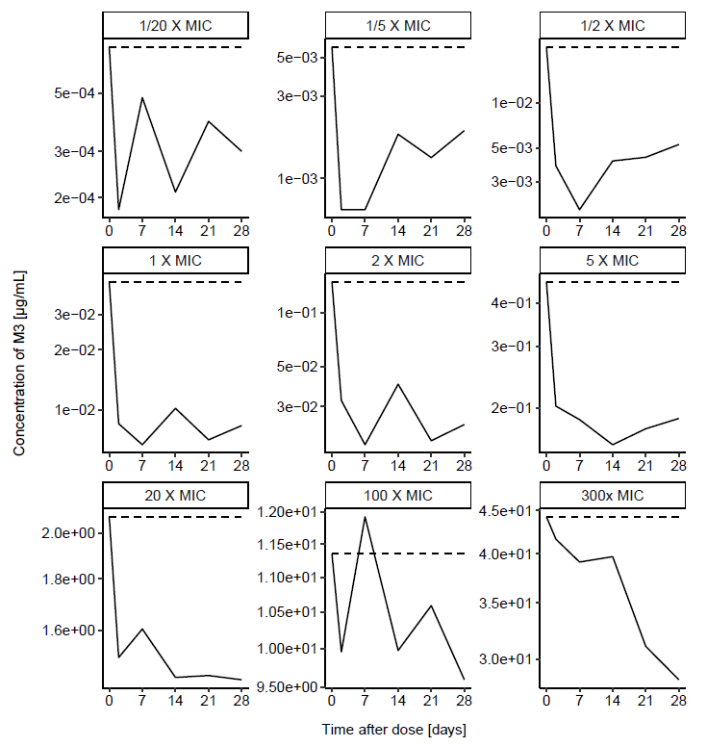


**Figure S10. Drug concentration of M12 in standard broth.** Relative concentration to time zero of M12 in standard medium for the time kill assay, dotted line marks the baseline concentration. Below quantification limit marked on x-axis.


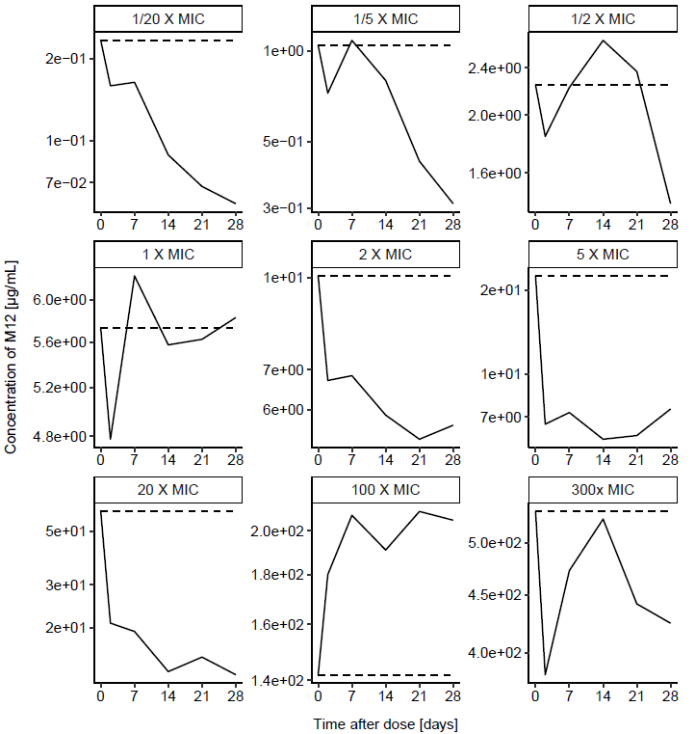


**Figure S11. Drug concentration of M12 in cholesterol broth.** Relative concentration to time zero of M12 in cholesterol medium for the time kill assay, dotted line marks the baseline concentration. Below quantification limit marked on x-axis.


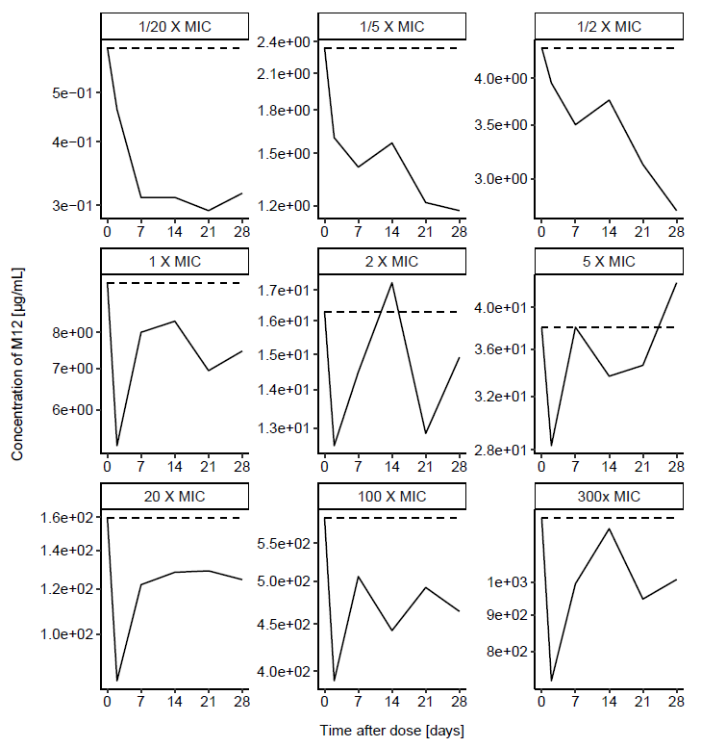


**Figure S12. Drug concentration of M12 in fatty acids broth.** Relative concentration to time zero of M12 in fatty acids medium for the time kill assay, dotted line marks the baseline concentration. Below quantification limit marked on x-axis.


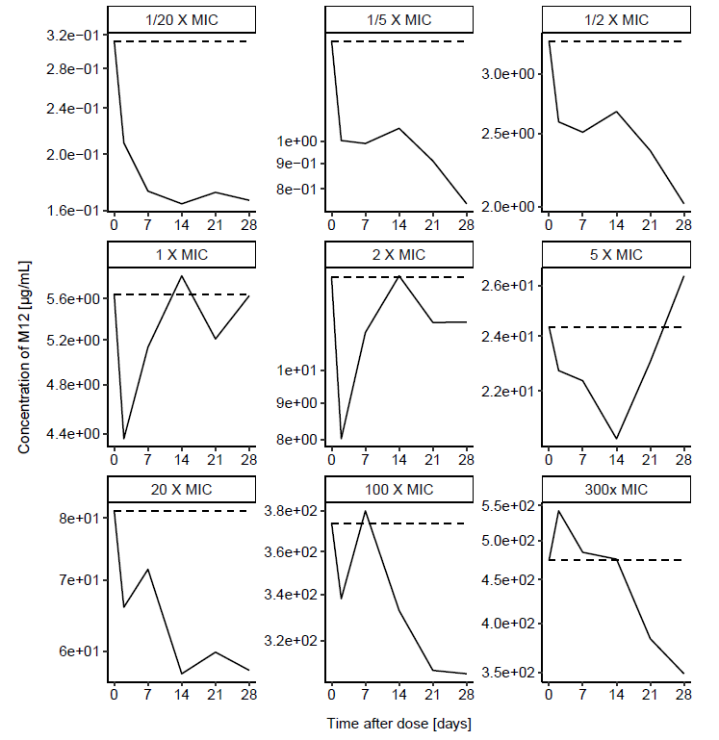


**Figure S13. Stability of TBAJ-587 M3 metabolite.** The recovery of TBAJ-587-M3 metabolite was evaluated in standard 7H9 + 0.5%BSA medium at 10x MIC, without bacteria, in glass and low binding polypropylene tubes over six days at 37°C.


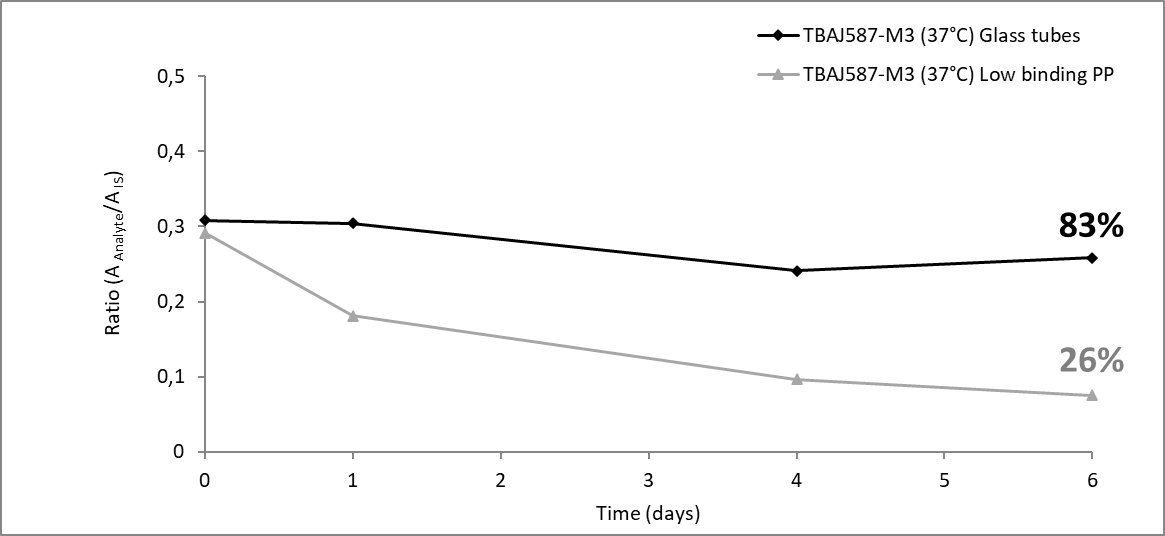


**Figure S14. Impact of tyloxapol on the stability of TBAJ-587.** Recovery of TBAJ-587 was evaluated over time at **(A)** high (1.6 mg/L) and **(B)** low (0.16 mg/L) concentrations in ST broth medium without and with 0.05 % tyloxapol in the absence of bacteria. The control drug propranolol was also evaluated in the same conditions as TBAJ-587 at **(C)** high (1.6 mg/L) and **(D)** low (0.16 mg/L) concentrations.

**
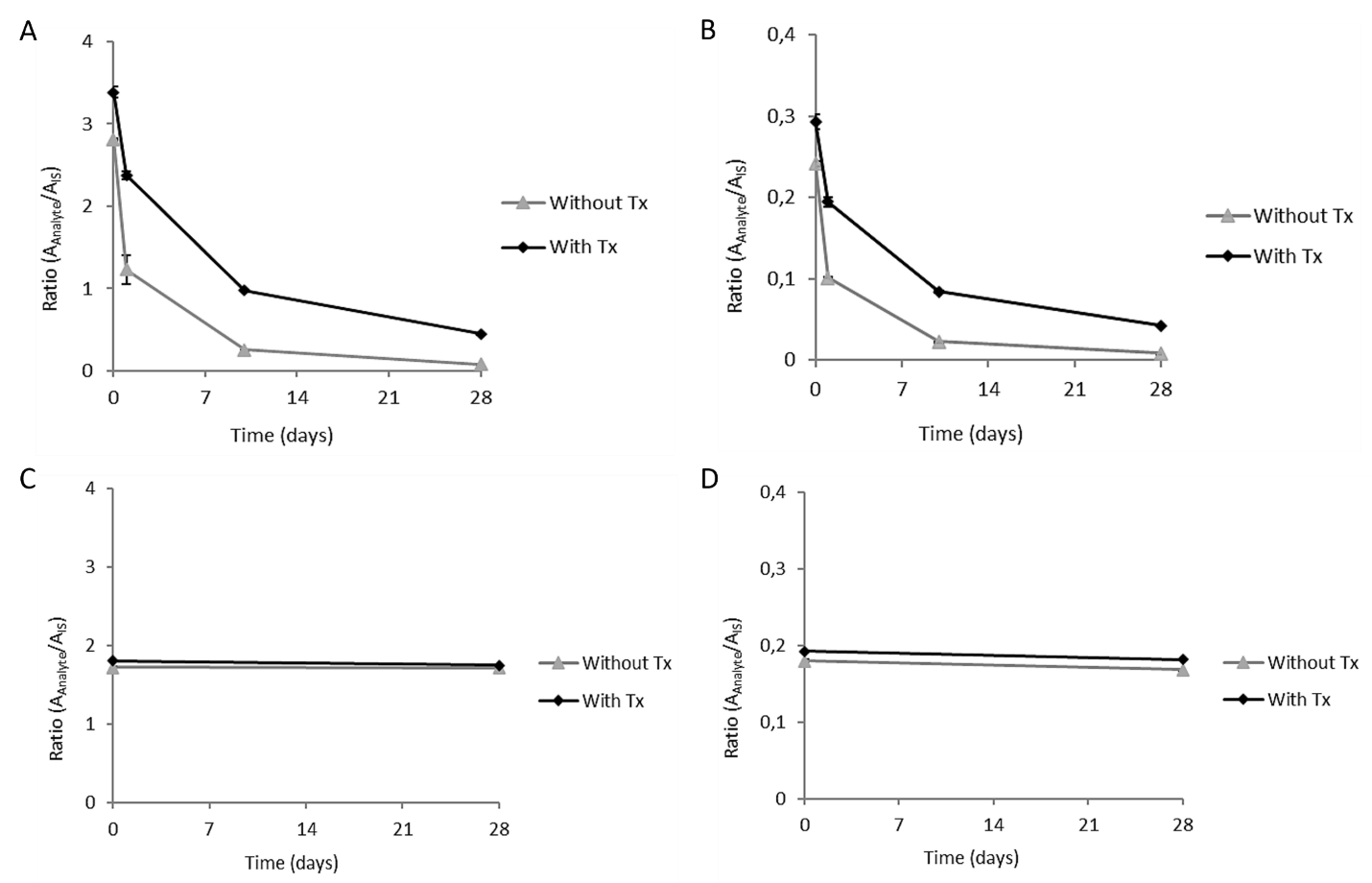
**
